# Development of atrial fibrillation following trauma increases short term risk of cardiovascular events

**DOI:** 10.1515/jom-2020-0260

**Published:** 2021-03-10

**Authors:** Sean P. Nassoiy, Robert H. Blackwell, McKenzie Brown, Anai N. Kothari, Timothy P. Plackett, Paul C. Kuo, Joseph A. Posluszny

**Affiliations:** Department of Surgery, One: MAP Surgical Analytics, Loyola University Medical Center, Maywood, IL, USA

**Keywords:** atrial fibrillation, cardiopulmonary medicine, cerebrovascular accident, emergency medicine, hospitalization, myocardial infarction, stroke, trauma

## Abstract

**Context:**

New onset atrial fibrillation (AF) is associated with poor outcomes in several different patient populations.

**Objectives:**

To assess the effect of developing AF on cardiovascular events such as myocardial infarction (MI) and cerebrovascular accident (CVA) during the acute index hospitalization for trauma patients.

**Methods:**

The Healthcare Cost and Utilization Project State Inpatient Databases for California and Florida were used to identify adult trauma patients (18 years of age or older) who were admitted between 2007 and 2010. After excluding patients with a history of AF and prior history of cardiovascular events, patients were evaluated for MI, CVA, and death during the index hospitalization. A secondary analysis was performed using matched propensity scoring based on age, race, and preexisting comorbidities.

**Results:**

During the study period, 1,224,828 trauma patients were admitted. A total of 195,715 patients were excluded for a prior history of AF, MI, or CVA. Of the remaining patients, 15,424 (1.5%) met inclusion criteria and had new onset AF after trauma. There was an associated increase in incidence of MI (2.9 vs. 0.7%; p<0.001), CVA (2.6 vs. 0.4%; p<0.001), and inpatient mortality (8.5 vs. 2.1%; p<0.001) during the index hospitalization in patients who developed new onset AF compared with those who did not. Cox proportional hazards regression demonstrated an increased risk of MI (odds ratio [OR], 2.35 [2.13–2.60]), CVA (OR, 3.90 [3.49–4.35]), and inpatient mortality (OR, 2.83 [2.66–3.00]) for patients with new onset AF after controlling for all other potential risk factors.

**Conclusions:**

New onset AF in trauma patients was associated with increased incidence of myocardial infarction (MI), cerebral vascular accident (CVA), and mortality during index hospitalization in this study.

Atrial fibrillation (AF) is the most common cardiac arrhythmia [1–4]. It is particularly common among critically ill patients; nearly a quarter (27%) of the 10,836 patients in a 2016 study experienced AF [5]. New onset AF is associated with significant morbidity and mortality [1, 5, 6]. In a secondary analysis of the Reasons for Geographic and Racial Differences in Stroke cohort, mortality for new onset AF was twice as high in patients who developed AF when compared with patients who did not [7]. New onset AF also has been associated with poor cardiovascular outcomes in patients who have undergone noncardiac surgery, gastrectomy, and radical cystectomy [8–10]. Specifically, AF has been shown to increase the risk of stroke between three and five times [1, 7], while stroke and AF have been associated with longer hospital stays, greater mortality, and increased hospital costs [1]. However, the risks remain unclear in patients who develop new onset AF following trauma. Therefore, further investigation in this patient population is warranted.

The objective of this study was to determine whether the development of new onset AF in trauma patients conferred higher risk for cardiovascular events during the same admission. We investigated cardiovascular morbidity and mortality for trauma patients who developed AF – specifically, the rates of myocardial infarction (MI) and cerebral vascular accident (CVA) during index hospitalization. We studied patients with no prior diagnosis of AF to determine whether those patients were at increased risk for MI, CVA, or death when compared with a risk matched cohort of patients who did not develop AF.

## Methods

This study was reviewed and deemed exempt by the institutional review board at Loyola University Medical Center due to its use of publicly available data that lacked patient identifiers.

We performed a retrospective review using the Healthcare Cost and Utilization Project State Inpatient Database (HCUP-SID) for California and Florida for the years 2006 through 2011. All patients 18 years of age and older with a traumatic injury admitted to the hospital from 2007 to 2010 were included. Patients of interest were identified based on the use of the International Classifications of Disease, ninth Revision (ICD-9) E codes 800–998. AF, MI, coronary artery disease (CAD), CVI, and other conditions were identified using ICD-9 codes previously described by Blackwell et al. [10]. Conditions were differentiated between preexisting or occurring during hospitalization. Patients with a prior history of AF were excluded to assess the effect of new onset AF. Patients with a prior history of MI or CAD were excluded, as a history of prior cardiac ischemia may have affected the development of new onset AF or subsequent cardiac events. Patients with a prior history of CVA or transient ischemic attack (TIA) were excluded to ensure that only new onset CVAs were detected.

Patient demographics abstracted from the database included age, gender, race/ethnicity, and primary insurance provider. Medical comorbidities abstracted included obesity, diabetes mellitus, hypertension, congestive heart failure, valvular heart disease, chronic renal failure, MI, CAD, cerebrovascular accident, and TIA. Charlson Comorbidity Index and CHA_2_DS_2_VASc (congestive heart failure, hypertension, age, diabetes, previous stroke/transient ischemic attack, vascular disease, and sex) scores were calculated for each patient to allow for comparison of comorbidities and baseline stroke risk [11, 12]. The category of trauma was determined based on the ICD-9 E code. HCUP-SID categories of trauma included both mechanism of injury (transportation, falls, nature, legal intervention, and war) and intention (suicide/self-inflicted, accidentally-inflicted, and homicidal/purposefully-inflicted). The occurrence of AF, MI, CVA, and death during the index hospitalization were also recorded.

Descriptive statistics are presented as mean ± standard deviation (SD) or n (%). Independent t-test and chi-square test were used for univariate analysis comparing patients with and without new onset AF. Univariate and multivariate logistic regression models were fit to assess the odds ratio of new onset AF on the outcomes of interest (MI, CVA, mortality). The multivariate logistic regression model adjusted the odds ratio for age, race, gender, primary insurance provider, Charlson score, CHA_2_D_2_VASc, and preexisting medical diagnoses to control for potential confounding variables. Results are presented as odds ratios with 95% confidence interval (CI).

## Results

**Figure 1:**
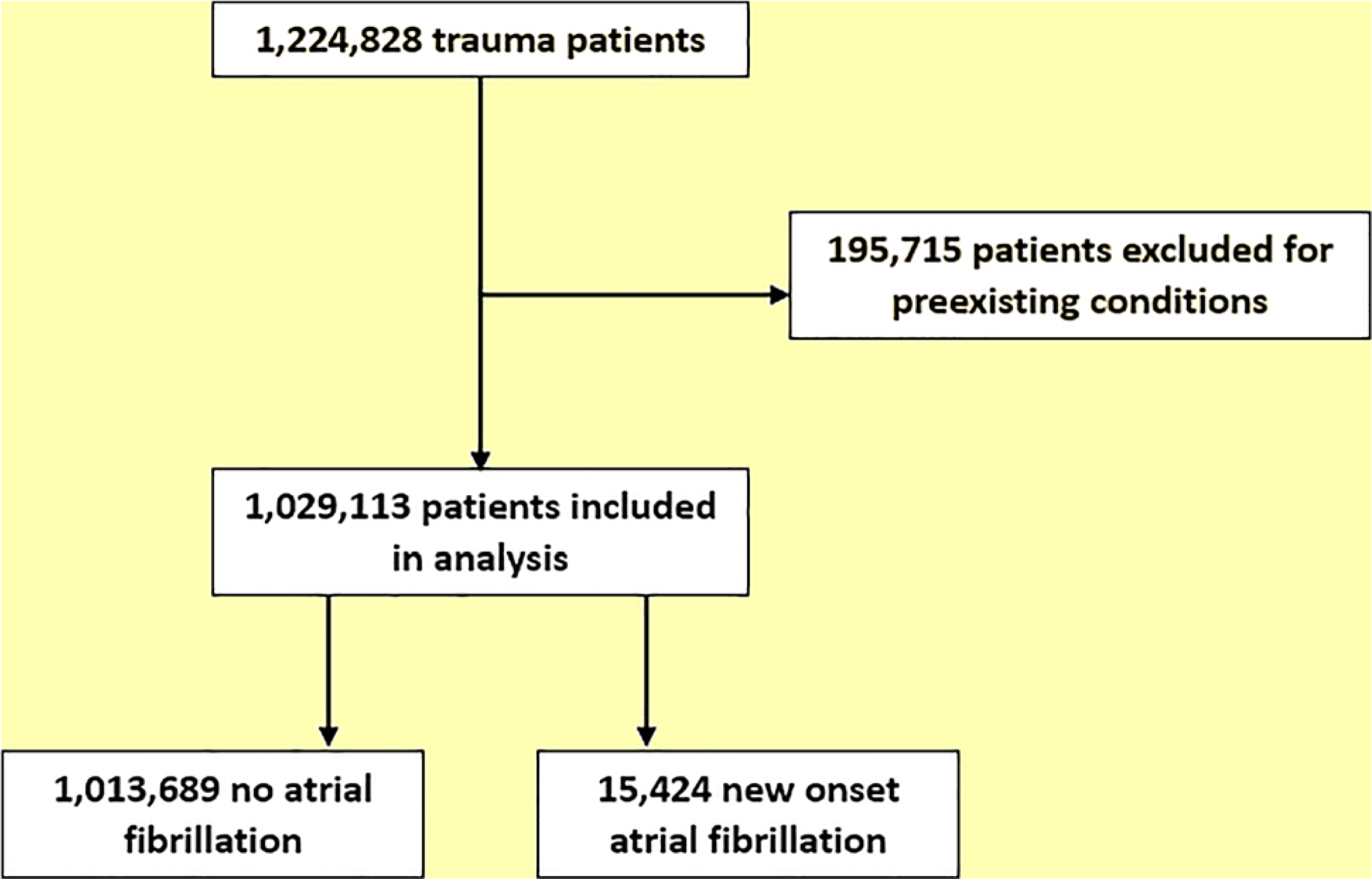
Consolidated standards of reporting trials (CONSORT) diagram depicting the flow of patients through this study.

Figure 1 shows the flow of patients through this retrospective study. Over a five-year period, 1,224,828 trauma patients were admitted to a hospital in California or Florida. Of these patients, 195,715 (16.0%) had a prior history of AF, MI, CAD, CVA, or TIA and were excluded from further analysis. Of the remaining 1,029,113 patients with no history of AF (“never AF”), 15,424 (1.5%) developed new onset AF. Patients who developed new onset AF were more likely than those who did not develop AF (“no AF”) to be older (mean age, 77 years for new onset AF vs. 60 years for no AF; p<0.001), and to carry Medicare or Medicaid as their primary insurance provider (83.4% for new onset AF vs. 63.9% for no AF; p<0.001) (Table 1). Men comprised a larger percentage of new onset AF patients compared with women (52.0 vs. 48.0%, respectively; p<0.001) and women comprised a larger percentage of the no AF group compared with men (52.2 vs. 47.8%, respectively; p<0.001). Patients with new onset AF were more likely to have preexisting comorbidities of obesity (11.1% for new onset AF vs. 7.3% for no AF; p<0.001), diabetes mellitus (23.1% for new onset AF vs. 15.4%; for no AF; p<0.001), hypertension (72.1% for new onset AF vs. 47.2% for no AF; p<0.001), congestive heart failure (16.0% for new onset AF vs. 5.0% for no AF; p<0.001), valvular heart disease (10.7% for new onset AF vs. 3.7% for no AF; p<0.001), chronic renal failure (17.3% for new onset AF vs. 9.2% for no AF; p<0.001), and peripheral vascular disease (13.5% for new onset AF vs. 5.3% for no AF; p<0.001). They had a higher mean Charlson Comorbidity Index score (2 for new onset AF vs. 0 for no AF; p<0.001) and CHA_2_DS_2_VASc score (3 for new onset AF vs. 2 for no AF; p<0.001). Finally, the patients who developed new onset AF were more likely to have sustained transportation related trauma (72.3% for new onset AF vs. 61.1% for no AF; p<0.001). All other mechanisms of injury displayed a negative association with the development on new onset AF. All injury intention categories showed a negative association to the development of new onset AF.

Myocardial infarction occurred in 7,675 patients (0.7%) after injury. The incidence was significantly increased in patients who developed AF (445; 2.9%) compared with no AF patients (7,230; 0.7%; p<0.001). On univariate analysis, the risk of MI was associated with new onset AF (OR, 4.14[CI, 3.75–4.55]), congestive heart failure (OR 3.04 [CI, 2.84–3.24]), hypertension (OR, 2.96 [CI, 2.82–3.11]), peripheral vascular disease (OR, 2.09 [CI, 1.94–2.24]), valvular heart disease (OR, 2.03 [CI, 1.87–2.33]), chronic renal failure (OR, 1.96 [CI, 1.84–2.09]), diabetes mellitus (OR, 1.84 [CI, 1.76–1.94]), and obesity (OR, 1.03 [CI, 1.23–1.43]) (Table 2). The risk of MI was also associated with increased Charlson Comorbidity Index and CHA_2_DS_2_VASc. On adjusted multivariate logistic regression, an injury mechanism of war was the greatest risk factor for MI (OR, 5.89 [CI, 1.37–25.2]), followed by new onset AF (OR, 2.35 [CI, 2.13–2.60]).

Cerebrovascular accidents occurred in 4,242 patients (0.4%) after injury. The incidence was significantly increased in patients who developed AF (396; 2.6% of new onset AF) compared with no AF patients (3,846; 0.4% of no AF patients; p<0.001). On univariate analysis, the risk of CVA was associated with new onset AF (OR, 6.92 [CI, 6.23–7.68]), peripheral vascular disease (OR, 3.32 [CI, 3.05–3.60]), congestive heart failure (OR, 2.88 [CI, 2.64–3.15]), chronic renal failure (OR, 2.54 [CI, 2.36–2.75]), hypertension (OR, 2.53 [CI, 2.37–2.70]), valvular heart disease (OR, 2.21 [CI, 1.98–2.47]), diabetes mellitus (OR, 1.86 [CI, 1.73–1.99]), and obesity (OR, 1.47 [CI, 1.22–1.62]) (Table 3). The risk of CVA was also independently associated with increased Charlson Comorbidity Index and CHA_2_DS_2_VASc. On adjusted multivariate logistic regression, new onset AF was the strongest risk factor for CVA during hospitalization (OR 3.90 [CI, 3.49–4.35]), followed by hypertension (OR 1.27 [CI, 1.16–1.38]).

Death occurred in 21,202 patients (2.1%). The incidence was significantly increased in patients who developed AF (1,312; 8.5%) compared with no AF patients (20,890 [2.1%]; p<0.001). On univariate analysis, the risk of inpatient mortality was associated with new onset AF (OR, 4.42 [CI, 4.17–4.68]), congestive heart failure (OR 3.70 [CI, 3.56–3.84]), chronic renal failure (OR, 2.68 [CI, 2.59–2.78]), peripheral vascular disease (OR, 2.22 [CI, 2.13–2.32]), valvular heart disease (OR, 1.68 [CI, 1.59–1.78]), diabetes mellitus (OR, 1.27 [CI, 1.23–1.31]), and hypertension (OR, 1.12 [CI, 1.09–1.15]) (Table 4). The risk of inpatient mortality was also independently associated with increased Charlson Comorbidity Index and CHA_2_DS_2_VASc. On adjusted multivariate logistic regression, the strongest risk factor for inpatient mortality was accidentally inflicted trauma (OR, 3.69 [CI, 3.11–4.39]), followed by new onset AF (OR, 2.83 [CI, 2.66–3.00]).

## Discussion

While a history of AF is common [3, 4], new onset AF is a relatively less frequent finding following injury. Prior single institution studies have reported an AF incidence of 4–7% [2, 13, 14]. In contrast, studies utilizing administrative databases, such our present study, have offered a more conservative AF incidence of 1.5–2.5% [3, 15]. The two- to threefold difference in incidence could be reflective of the limitations of administrative databases [16]. Notwith-standing the variability in incidence, new onset AF occurs with enough frequency that better understanding of the problem and its implications for trauma patients is warranted.

Multiple factors have the potential to contribute to the development of AF and, for trauma patients, these can be grouped into two overarching categories: preexisting risk factors and risk factors associated with the injury and its management. A partial list of preexisting risk factors includes advancing age, male sex, coronary artery disease, hypertension, heart failure, valvular heart disease, diabetes, obesity, and chronic kidney disease [17–19]. Not surprisingly, all of these risk factors were found at greater frequency in trauma patients that developed new onset AF in our study compared with those who did not. Preexisting obesity, diabetes, and hypertension resulted in 1.5-fold increase in new onset AF. Chronic renal failure resulted in a 1.9-fold increase in new onset AF, while peripheral vascular disease resulted in a 2.5-fold increase, valvular heart disease in a 2.9-fold increase, and chronic heart failure resulted in a 3.2-fold increase. Thus, the contribution of preexisting medical conditions to the development on new onset AF should not be overlooked. It is also likely that some patients may have had paroxysmal AF that was undiagnosed prior to their hospitalization. A point-prevalence study suggested that 1% of the adult population falls into this category [20]; however, it is impossible to determine how many patients from the present study had already been affected by undiagnosed AF.

Risk factors associated with the injury itself include higher injury severity scores, blunt trauma vs. penetrating trauma, intracranial hemorrhage, and the presence of hemorrhagic shock [13, 14, 21]. Risk factors associated with the management of injured patients include increased transfusion of blood products, increased volume of fluid resuscitation, and the utilization of exogenous catecholamines [13]. In keeping with this, transportation and falls (both predominantly blunt mechanisms) were the two most common categories of trauma for patients with new onset AF in our study. Traditionally, arrhythmias after trauma (including but are not limited to AF) have been conceptualized and discussed in association with blunt cardiac injury [22, 23]. However, this focus is likely too narrow and instead, blunt trauma should be viewed as the risk factor regardless of cardiac involvement. Supporting this conclusion, Motz et al. [21] examined 258 trauma patients with AF and, of the 127 patients who had an echocardiogram, none demonstrated evidence of blunt cardiac injury. It is plausible that the increased risk of AF following blunt trauma may be related less to potential anatomic or physiologic differences in blunt vs. penetrating trauma, and rather, blunt trauma may be a confounder for other, more relevant risk factors. For example, blunt trauma patients tend to be older than penetrating trauma patients and the incidence of comorbidities increases with age [24, 25]. The mechanism of injury became much less significant on multiviariate analysis when age and comorbidities were also accounted for in our study. Finally, trauma induces a significant sympathetic response [26]. This increase in autonomic activity can be a predisposing factor to AF [27] and provides an additional mechanism linking trauma to new onset AF.

Patients who developed new onset AF after trauma in our study were found to have significantly increased risk of MI and CVA during the index hospitalization on univariate analysis. This is similar to results found in Lai et al.’s analysis of the Taiwanese National Health Insurance database [15]. In their analysis of over 1 million patients admitted to the hospital for traffic-related injuries, AF was associated with an eightfold increase in the incidence of MI and ninefold increase in CVA. However, the significant difference in comorbidities between patients who developed AF and those who did not mandates more than a univariate analysis. On multivariate analysis, after matching for cardiovascular comorbidities, new onset AF remained the one of the greatest risk factors associated with MI and CVA.

In the absence of trauma, AF is associated with up to a 3% rate of MI per year [28, 29], which represents a 1.5-fold increase risk compared with individuals without AF [29, 30]. In contrast, in our present study of trauma patients, new onset AF was associated with 2.9% risk of MI during hospitalization and, on multivariate logistic regression, the relative risk showed a 2.3-fold increase. While the risk between trauma and nontrauma patients with AF appear relatively similar, it is important to consider that the nontrauma risk is calculated over a year, whereas for trauma patients, it is only for the duration of admission (typically less than 30 days). As such, this is an acutely heightened time of risk for trauma patients. The exact mechanism by which AF leads to MI is not completely understood. Three predominant mechanisms have been proposed: inflammation leading to a prothrombotic state, tachyarrhythmias causing a mismatch between supply and demand, and direct coronary thromboembolism [28]. The first two of these mechanisms can be further aggravated by trauma. There is a well characterized inflammatory and prothrombotic state that develops after injury [31, 32]. Additionally, supply-demand mismatch is a key component of hemorrhagic shock and a driving factor for resuscitation strategies [33]. The interaction of these factors in the setting of AF requires further study to more clearly establish the mechanism and degree to which trauma and AF increases the risk of MI.

Strokes occur in approximately 1–2% of patients with AF every year [11, 34]. For patients with new onset AF, the risk increases following surgery or major illness [1, 9, 10, 34, 35]. In keeping with this trend, the incidence of CVA for trauma patients with new onset AF in our study was 2.6%. As with MI, trauma patients with new onset AF had a higher relative risk of CVA than patients who never experienced AF in our study. Similarly, this trend remained even after controlling for the significant differences in cardiovascular comorbidities between the two populations.

Mortality was significantly increased for patients with new onset AF. Even after controlling for the differences in comorbidities between patients with and without new onset AF, AF remained a predictor of mortality. In the present study, new onset AF was associated with a doubled risk of mortality. This was similar to what has been reported by other groups that researched trauma patients [13–15], patients with sepsis [6], and patients in critical care [36, 37]. While a potential mechanism can be proposed whereby AF leads to MI and CVA, AF is more likely a marker for risk of mortality rather than a proximate cause of mortality in the trauma population. In an analysis from Hadjizacharia et al. [14] of trauma patients with atrial arrhythmias, the presence of an atrial arrhythmia doubled the risk of mortality during hospitalization, but only 7% of the deaths were from a cardiovascular origin [14]. Given the low percentage of deaths attributed to a cardiovascular cause, it is less likely that mortality can be directly attributed to AF.

### Limitations

The limitations of our study warrant acknowledgement. As previously noted, research utilizing an administrative database is constrained by the breadth and accuracy of the data collected [16]. For example, because injury was identified using HCUP-SID definitions that based on ICD-9 E codes, our data does not specify injury beyond the mechanism. Regions injured, number of regions injured, and severity of injury have been shown to influence rates of new onset AF and outcomes [13–15]. However, these important variables were not included in the database and were unable to be accounted for in the multivariate logistic regression model. Additionally, the categories of trauma reported in the HCUP-SID were extremely broad and there was overlap between more traditional categories of blunt and penetrating injury within the same HCUP definition. As such, we urge caution in interpreting our results as they relates to mechanism of injury.

Atrial fibrillation was treated as a binary event. While this was done due to the limitation of the HCUP-SID (which only records the absence or presence of atrial fibrillation during admission), it fails to account for the incredible complexity of AF. The origin (valvular vs. nonvalvular) and burden (paroxysmal vs. nonparoxysmal, longest duration, number of episodes over a defined period of time, and portion of time in AF over a defined period of time) influence outcomes [38–40]. Future studies should strive to evaluate the impact of these variables in the trauma population in order to provide a more complete analysis.

Last, we were unable to account for the use of anticoagulation or other therapies that may have influenced the risk of heart attack or stroke, as those data points are not included in HCUP-SID. While guidelines recommend the use of anticoagulation for patients with an appropriate CHA_2_DS_2_-VASc score, the recommendation is balanced by the notion that risk of bleeding must also be taken into consideration [41]. It is plausible to think that treating physicians may have considered the risk of bleeding too great and not have started anticoagulation, thereby increasing the risk of a cardiovascular event. This hypothesis needs further investigation.

## Conclusions

In conclusion, new onset AF in trauma patients was associated in our study with an increased risk of in-hospital MI, CVA, and mortality independent of other known risk factors. New onset AF was a stronger risk factor for MI and CVA than other traditional cardiovascular risk factors. Trauma patients who develop AF should be recognized to have increased risk of cardiovascular events and mortality, so that rational treatment strategies can be better tailored to supporting this population during hospitalization.

## Figures and Tables

**Table 1:** Univariate analysis of new onset atrial fibrillation in trauma patients based on patient demographics, select medical comorbidities, and type of trauma.

		New onset atrial fibrillation		p-value
		No (n=1,013,689) n (%)	Yes (n=15,424) n (%)	

**Demographics**				
Age, years, median, IQR		60 (40–78)	77 (68–84)	<0.001
Sex	Men	484,808 (47.8)	8,018 (52.0)	<0.001
	Women	528,881 (52.2)	7,406 (48.0)	
Primary insurance provider	Medicare/Medicaid	592,961 (63.9)	12,528 (83.4)	<0.001
	Private insurance	245,330 (26.4)	2,214 (14.7)	
	Self pay/unknown	89,692 (9.7)	285 (1.9)	

**Medical comorbidities**				
Obesity		73,678 (7.3)	1,707 (11.1)	<0.001
Diabetes mellitus		156,405 (15.4)	3,560 (23.1)	<0.001
Hypertension		478,547 (47.2)	11,113 (72.1)	<0.001
Congestive heart failure		50,727 (5.0)	2,462 (16.0)	<0.001
Valvular heart disorder		38,258 (3.7)	1,648 (10.7)	<0.001
Chronic renal failure		83,009 (9.2)	2,675 (17.3)	<0.001
Peripheral vascular disease		53,646 (5.3)	2,086 (13.5)	<0.001
Charlson comorbidity index, median, IQR		0 (0–2)	2 (1–3)	<0.001
CHA_2_DS_2_VASc score, median, IQR		2 (1–3)	3 (2–4)	<0.001

Trauma type*				
Transportation		619,110 (61.1)	11,152 (72.3)	<0.001
Falls		410,229 (40.5)	5,948 (38.6)	<0.001
Nature		18,650 (1.8)	34 (0.2)	<0.001
Accident		125,421 (12.4)	1,057 (6.9)	<0.001
Suicide/self inflicted		72,545 (7.2)	118 (0.8)	<0.001
Homicide/purposefully inflicted		34,850 (3.4)	80 (0.5)	<0.001
Legal intervention**		1,126 (0.1)	−(0.01)	<0.001
Accidentally inflicted		2,632 (0.3)	17 (0.1)	<0.001
War		55 (0.01)	0 (0)	0.4

* Percentages add to greater than 100%, as patients may carry more than one trauma diagnosis.

** Data censored given restrictions in publishing values of n<10. CHA_2_DS_2_VASc, congestive heart failure, hypertension, age, diabetes, previous stroke/transient ischemic attack, vascular disease, and sex; IQR, interquartile range.

**Table 2:** Univariate and multivariate logistic regression for myocardial infarction in trauma patients based on patient demographics, select medical comorbidities, type of trauma, and presence of new onset atrial fibrillation.

		Univariate OR (95% CI)	p-value	Multivariate OR (95% CI)	p-value

**Demographics**					
Age		1.03 (1.03–1.03)	<0.001	1.02 (1.01–1.02)	<0.001
Sex	Men relative to women	0.98 (0.94–1.03)	0.4	-	-
Primary insurance provider	Medicare/Medicaid	2.60 (2.43–2.78)	<0.001	1.24 (1.15–1.34)	<0.001
	Private insurance	Referent		Referent	
	Self pay/unknown	0.71 (0.61–0.81)	<0.001	1.00 (0.87–1.15)	0.9

**Medical comorbidities**					
Obesity		1.33 (1.23–1.43)	<0.001	1.15 (1.06–1.25)	0.001
Diabetes mellitus		1.84 (1.76–1.94)	<0.001	1.03 (0.97–1.10)	0.3
Hypertension		2.96 (2.82–3.11)	<0.001	1.60 (1.50–1.70)	<0.001
Congestive heart failure		3.04 (2.84–3.24)	<0.001	1.22 (1.23–1.43)	<0.001
Valvular heart disorder		2.03 (1.87–2.22)	<0.001	1.14 (1.23–1.44)	0.005
Chronic renal failure		1.96 (1.84–2.09)	<0.001	0.70 (0.65–0.74)	<0.001
Peripheral vascular disease		2.09 (1.94–2.24)	<0.001	1.02 (0.94–1.11)	0.7
Charlson comorbidity index		1.27 (1.26–1.28)	<0.001	1.23 (1.22–1.24)	<0.001
CHA_2_DS_2_VASc score		1.46 (1.44–1.48)	<0.001	1.01 (0.98–1.04)	0.5

**Trauma type**					
Transportation		0.92 (0.88–0.96)	<0.001	0.93 (0.88–0.99)	0.02
Falls		1.66 (1.58–1.73)	<0.001	1.08 (1.01–1.14)	0.02
Nature		0.42 (0.32–0.54)	<0.001	0.82 (0.62–1.07)	0.1
Accident		0.83 (0.78–0.90)	<0.001	1.03 (0.94–1.12)	0.6
Suicide/self inflicted		0.40 (0.35–0.46)	<0.001	0.96 (0.82–1.12)	0.6
Homicide/purposefully inflicted		0.30 (0.24–0.37)	<0.001	0.88 (0.68–1.15)	0.3
Legal intervention		0.71 (0.32–1.59)	0.4	-	-
Accidentally inflicted		0.76 (0.56–1.26)	0.3	-	-
War		5.02 (1.22–30.61)	0.02	5.89 (1.37–25.2)	0.02
New onset atrial fibrillation		4.14 (3.75–4.55)	<0.001	2.35 (2.13–2.60)	<0.001

CHA_2_DS_2_VASc, congestive heart failure, hypertension, age, diabetes, previous stroke/transient ischemic attack, vascular disease, and sex; CI, confidence interval; OR, odds ratio.

**Table 3:** Univariate and multivariate logistic regression for cerebral vascular accident in trauma patients based on patient demographics, select medical comorbidities, type of trauma, and presence of new onset atrial fibrillation.

		Univariate OR (95% CI)	p-value	Multivariate OR (95% CI)	p-value

**Demographics**					
Age		1.02 (1.02–1.02)	<0.001	1.0 (0.99–1.00)	0.4
Sex	Men relative to women	0.99 (0.93–1.05)	1.0	-	-
Primary insurance provider	Medicare/Medicaid Private insurance	1.71 (1.58–1.85) Referent	<0.001	0.94 (0.85–1.02) Referent	0.1
	Self pay/unknown	0.54 (0.45–0.64)	<0.001	0.79 (0.66–0.94)	0.01

**Medical comorbidities**					
Obesity		1.47 (1.22–1.62)	<0.001	1.18 (1.06–1.31)	0.002
Diabetes mellitus		1.86 (1.73–1.99)	<0.001	0.79 (0.73–0.86)	<0.001
Hypertension		2.53 (2.37–2.70)	<0.001	1.27 (1.16–1.38)	<0.001
Congestive heart failure		2.88 (2.64–3.15)	<0.001	0.90 (0.82–1.00)	0.055
Valvular heart disorder		2.21 (1.98–2.47)	<0.001	1.36 (1.21–1.52)	<0.001
Chronic renal failure		2.54 (2.36–2.75)	<0.001	0.70 (0.64–0.76)	<0.001
Peripheral vascular disease		3.32 (3.05–3.60)	<0.001	1.18 (0.64–0.76)	0.001
Charlson comorbidity index		1.37 (1.36–1.38)	<0.001	1.22 (1.21–1.34)	<0.001
CHA_2_DS_2_VASc score		1.47 (1.45–1.50)	<0.001	1.23 (1.19–1.27)	<0.001

**Trauma type**					
Transportation		1.56 (1.46–1.66)	<0.001	1.24 (1.15–1.33)	<0.001
Falls		0.96 (0.90–1.02)	0.1	-	-
Nature		0.20 (0.12–0.33)	<0.001	0.36 (0.21–0.61)	<0.001
Accident		0.60 (0.54–0.67)	<0.001	0.70 (0.62–0.79)	<0.001
Suicide/self inflicted		0.20 (0.15–0.25)	<0.001	0.47 (0.36–0.61)	<0.001
Homicide/purposefully inflicted		0.42 (0.33–0.54)	<0.001	1.05 (0.79–1.40)	0.4
Legal intervention		-	-	-	-
Accidentally inflicted		0.18 (0.05–0.73)	0.02	0.32 (0.08–1.27)	0.1
War		-	-	-	-
New onset atrial fibrillation		6.92 (6.23–7.68)	<0.001	3.90 (3.49–4.35)	<0.001

CHA_2_DS_2_VASc, congestive heart failure, hypertension, age, diabetes, previous stroke/transient ischemic attack, vascular disease, and sex; CI, confidence interval; OR, odds ratio.

**Table 4:** Univariate and multivariate logistic regression for death in trauma patients based on patient demographics, select medical comorbidities, type of trauma, and presence of new onset atrial fibrillation.

		Univariate OR (95% CI)	p-value	Multivariate OR (95% CI)	p-value

**Demographics**					
Age		1.01 (1.01–1.01)	<0.001	1.01 (1.01–1.01)	<0.001
Sex	Men relative to women	1.50 (1.46–1.54)	<0.001	1.33 (1.28–1.37)	<0.001
Primary insurance provider	Medicare/Medicaid	1.26 (1.22–1.31)	<0.001	0.83 (0.80–0.86)	<0.001
	Private insurance	Referent		Referent	
	Self pay/unknown	1.02 (0.97–1.08)	0.4	1.11 (1.05–1.18)	<0.001

**Medical comorbidities**					
Obesity		0.94 (0.89–0.99)	0.03	0.85 (0.80–0.90)	<0.001
Diabetes mellitus		1.27 (1.23–1.31)	<0.001	0.81 (0.77–0.84)	<0.001
Hypertension		1.12 (1.09–1.15)	<0.001	0.62 (0.60–0.65)	<0.001
Congestive heart failure		3.70 (3.56–3.84)	<0.001	1.94 (1.85–2.03)	<0.001
Valvular heart disorder		1.68 (1.59–1.78)	<0.001	1.07 (1.01–1.13)	0.03
Chronic renal failure		2.68 (2.59–2.78)	<0.001	1.35 (1.30–1.41)	<0.001
Peripheral vascular disease		2.22 (2.13–2.32)	<0.001	1.11 (1.05–1.17)	<0.001
Charlson comorbidity index		1.24 (1.23–1.24)	<0.001	1.16 (1.15–1.17)	<0.001
CHA_2_DS_2_VASc score		1.28 (1.27–1.29)	<0.001	1.14 (1.11–1.16)	<0.001

**Trauma type**					
Transportation		1.21 (1.27–1.35)	<0.001	1.05 (1.01–1.09)	0.009
Falls		0.82 (0.80–0.84)	<0.001	0.69 (0.66–0.71)	<0.001
Nature		0.85 (0.77–0.95)	0.004	1.10 (0.98–1.24)	0.11
Accident		0.76 (0.72–0.79)	<0.001	0.72 (0.68–0.76)	<0.001
Suicide/self inflicted		0.65 (0.61–0.69)	<0.001	0.93 (0.86–1.00)	0.06
Homicide/purposefully inflicted		0.99 (0.91–1.06)	0.7	-	-
Legal intervention		0.94 (0.62–1.43)	0.8	-	-
Accidentally inflicted		3.33 (2.86–3.87)	<0.001	3.69 (3.11–4.39)	<0.001
War		2.62 (0.82–8.38)	0.12	-	-
New onset atrial fibrillation		4.42 (4.17–4.68)	<0.001	2.83 (2.66–3.00)	<0.001

CHA_2_DS_2_VASc, congestive heart failure, hypertension, age, diabetes, previous stroke/transient ischemic attack, vascular disease, and sex; CI, confidence interval; OR, odds ratio.
